# Introducing the Event‐Adjusted Rank Sum (EARS) Test: A Simple Approach to Survival Analysis Independent of Proportional Hazards

**DOI:** 10.1155/bmri/2142254

**Published:** 2025-11-18

**Authors:** Gustav Stålhammar

**Affiliations:** ^1^ Ocular Oncology Service and St. Erik Ophthalmic Pathology Laboratory, St. Erik Eye Hospital, Stockholm, Sweden; ^2^ Department of Clinical Neuroscience, Division of Eye and Vision, Karolinska Institutet, Stockholm, Sweden, ki.se

**Keywords:** EARS, event-adjusted rank sum test, proportional hazards, survival distributions, survival test

## Abstract

**Background:**

Survival analyses often violate the proportional hazards (PH) assumption, compromising the validity of widely used statistical tests such as the log‐rank test and Cox regression. To address this limitation, we introduce the event‐adjusted rank sum (EARS) test, a nonparametric method designed to provide robust time‐to‐event data analysis without relying on PH.

**Materials and Methods:**

The EARS test adjusts survival times by dividing each event time by the proportion of events within its respective group. These adjusted survival times are then compared using the Kruskal–Wallis test. To account for censoring, the resulting *p* value is adjusted based on the overall proportion of censored observations. We validated the EARS test through simulations involving 1000 cohorts with group sizes ranging from 50 to 1000 patients and censoring rates between 5% and 75%. Additionally, we compared the performance of EARS to the log‐rank test and restricted mean survival time (RMST) under both PH and non‐PH conditions using both simulated and clinical datasets.

**Results:**

In simulation studies, the EARS and log‐rank tests agreed in 96.6% of cases. Under the null hypothesis, the EARS test demonstrated a Type I error rate of 2% across two to five groups, slightly higher than the log‐rank test′s 1%. Power analyses revealed that EARS detected true differences in 63% of cases compared to 68% for the log‐rank test. In 1000 datasets violating the PH assumption, EARS identified significant differences in 94.4% of cases versus 98.0% for RMST, with both methods agreeing 96.4% of the time on null hypothesis rejection. Analyses of clinical cohorts further confirmed the reliability of the EARS test, showing consistent alignment with established tests in most scenarios.

**Conclusion:**

The EARS test offers a simple, nonparametric alternative for survival analysis that remains reliable across diverse conditions and varying censoring distributions. Its accessibility and robust performance make it a valuable tool for researchers and clinicians, especially in settings where the PH assumption is violated or advanced statistical software is unavailable. An R package implementing the EARS test is openly available.

## 1. Introduction

Time‐to‐event analyses, essential in clinical research, often involve comparing survival distributions between patient groups. Commonly observed phenomena in these analyses include crossing survival curves and varying effects over time in experimental arms, with certain groups experiencing higher event rates in early or late periods [1–7]. Such observations signify a deviation from proportional hazards (PH), challenging the foundational assumption of prevalent statistical tests like the log‐rank test, its derivatives, and Cox regression [8–10]. A review of 175 studies in notable journals revealed that one‐third exhibited survival curve convergence and nearly half demonstrated curve crossings, potentially leading to skewed results [3].

To address non‐PH scenarios, various alternatives have been proposed. These include subgroup analyses, introducing time‐varying coefficients for non‐PH covariates, combined hypotheses using Cox regressions with permutation test *p* values, and trial designs employing the MaxCombo test [11]. Restricted mean survival time (RMST) has been proposed as a robust alternative to the log‐rank test in scenarios where PH is violated. However, RMST requires the specification of a truncation time and often necessitates access to specialized software, which may limit its applicability in some clinical settings [12, 13]. Some methods, like the Gehan–Breslow–Wilcoxon, Peto–Peto, and Tarone–Ware tests, assign greater weight to early events to mitigate non‐PH influences, whereas the log‐rank test treats all time points equally [14].

Despite these advancements, such tests often require specialized statistical expertise. For example, the correct implementation of time‐varying Cox models demands careful model specification, verification of PH through Schoenfeld residuals, and interpretation of time‐dependent coefficients—procedures that can be technically challenging for clinicians and researchers without formal training in survival modeling. Similarly, some non‐PH methods, such as RMST or the MaxCombo test, require the selection of tuning parameters (e.g., truncation times or weight functions), which can influence results if chosen inappropriately. In addition, these approaches typically rely on statistical software packages that may not be readily available outside academic settings, and they often require access to complete individual patient data in a specialized format.

In response to these challenges, we introduce and validate the event‐adjusted rank sum (EARS) test, a nonparametric approach that simplifies survival analysis. It requires only a grouping variable, event indicator, and follow‐up time and can be implemented in widely available software, including Excel, without parameter tuning or model‐specific diagnostics. This combination of methodological simplicity and broad software accessibility makes it more feasible for use in diverse research environments, including settings where PH is uncertain or demonstrably violated. An R package implementing the EARS test is openly available at https://github.com/gustavstalhammar-ui/EARS.

## 2. Materials and Methods

The development of the EARS test was a process aimed at creating a statistical test for survival analysis that is accessible to clinicians and nonstatisticians, applicable across various time‐to‐event analyses, and independent of the PH assumption and parametric survival data distribution. This process utilized fictional survival data from the five cohorts, three of which were modeled on real‐life patient data, and two were specifically created for the study:

Cohort #1 (development cohort) consisted of 1000 patients in two groups. This cohort was designed to reflect PH over a simulated follow‐up of 100 months.

Cohort #2 (development cohort) consisted of 11,160 patients, similar to data from an integrated Cancer Genome Atlas (TCGA) clinical data resource [15]. These patients were diagnosed with 33 different types of cancer, and the data included patient sex and overall survival time. The median follow‐up time for censored was 2.2 years (interquartile range [IQR] 2.9).

Cohort #3 (validation cohort) consisted of 1412 patients, similar to data from a recent publication on prognostic factors related to the risk for developing metastases of uveal melanoma [16]. The data included tumor size and metastasis‐free survival time. The median follow‐up time for nonmetastatic patients was 3.8 years (IQR 3.5).

Cohort #4 (validation cohort) consisted of 190 patients, similar to a sample of Belgian patients with breast cancer [17]. The data included patient age and overall survival time. The median follow‐up time for censored was 13.6 years (IQR 4.6).

Cohort #5 (validation cohort) consisted of 1000 patients in two groups. This cohort was intentionally designed to depart from PH, with crossing survival curves over a simulated follow‐up of 100 months. The first group had all disease‐related events (e.g., death from acute myocardial infarction [AMI]) before 75 months, whereas the second group had all events after 50 months. Every seventh patient′s event was replaced with an event of a second type (e.g., death from causes other than AMI). The raw data for all cohorts are provided as supporting information files (available here).

No personal, sensitive, or other data on real‐life patients were collected from any cohort, including locations and dates of diagnosis or treatment, patient names, sex, ages, personal identity numbers, social security numbers, addresses, phone numbers, IP numbers, or photos. The used data had three columns, with the following headers: “Group,” “Time,” and “Event.” No additional data were collected. According to the Swedish Ethical Review Act, the requirement for ethical approval is waived if no personal or sensitive information is collected or processed, if no interventions are planned, if no biological materials are processed, and if the method does not aim to affect individuals, neither physically nor psychologically, and does not entail any risk of harm for the research subjects [18].

### 2.1. Calculation of Event‐Adjusted Survival Times

The EARS test focuses on the rank sum of times to the event of interest, considering that factors like the proportion of patients experiencing the event significantly influence survival outcomes. This test adjusts survival times based on the event proportion within a group. This adjustment is crucial for scenarios where a lower event proportion could still result in a shorter average time to event, potentially misleading the interpretation of survival outcomes. For instance, if 50% of patients in one group die from the event of interest, but only 1% do so in another group, the latter could have a shorter average time to event if these few events occur early. However, this does not indicate worse survival in the group with a lower event rate.

To implement this, the EARS test modifies survival time by dividing the follow‐up time for patients suffering from the event (e.g., death, disease progression, and complications after surgery) by the proportion of patients suffering from this event in the patient′s group. All patients, including those without the event, are included solely to determine this group‐specific proportion. For example, in a study on metastasis‐free survival in male and female lung cancer patients, if a male patient develops metastasis after 5 years, this time is divided by the proportion of males who develop metastasis. This adjustment factor is constant within each group but differs between groups. While it does not change the order of event times within a given group, it alters their position relative to event times from other groups when all are pooled for the Kruskal–Wallis ranking. This ensures that differences in event incidence are incorporated into the comparison, so a group with a lower event proportion but later events is not ranked equivalently to a group with a higher event proportion.

EARS outcomes are therefore influenced by both the proportion of patients experiencing the event in the compared groups and the timing of these events, but not by the absolute number of patients in each group, provided the number meets the minimum requirement of 5, as per the Kruskal–Wallis test.

The adjusted survival time (AST) is calculated as follows:

ASTi=TiPe,g,

where

• AST_*i*_ is the AST for the *i*th patient.
• *T*_*i*_ is the observed survival time for the *i*th patient.
• *P*_*e*,*g*_ is the proportion of patients experiencing the event in group *g*.

No AST is calculated for patients who do not experience the event of interest.

The null hypothesis (H_0_) assumes no difference in the distribution of survival times across groups, while the alternative hypothesis (H_1_) posits that at least one group′s survival distribution differs. The Kruskal–Wallis test evaluates these hypotheses by comparing the ranks of ASTs among groups.

Lastly, to account for censoring, a penalty is applied to the resulting Kruskal–Wallis *p* value by dividing it by one minus the overall proportion of censored patients (i.e., those who do not experience the event of interest but are alive at the last follow‐up or die from other causes). For example, if 50% of patients are censored, the Kruskal–Wallis *p* value is divided by 0.5 (1 − 0.5) to yield the final EARS *p* value. Similarly, if only 20% of patients are censored, the *p* value is divided by 0.8 (1 − 0.2), resulting in a smaller penalty for samples with lower censoring rates. This adjustment ensures that samples with higher proportions of censored data are appropriately penalized, reducing the likelihood of overinterpreting results from heavily censored datasets.

### 2.2. Statistical Analysis With the Kruskal–Wallis Test

The Kruskal–Wallis test, also known as the Kruskal–Wallis H test (named after William Kruskal and W. Allen Wallis), or one‐way ANOVA on ranks, is a nonparametric method used to determine whether samples come from the same distribution. This test is applicable for comparing two or more independent samples of equal or different sizes. It is an extension of the Mann–Whitney *U* test, which is limited to comparing only two groups [19]. When the Kruskal–Wallis test yields a significant result, it implies that at least one sample stochastically dominates another. However, the test does not specify which sample or samples demonstrate this stochastic dominance, nor does it indicate the number of pairs of groups where stochastic dominance is observed. In this study, the EARS test employs the Kruskal–Wallis test to compare the ASTs across groups. The average rank of each patient′s AST among all patients suffering from the event of interest in all compared groups is determined. These ranks are then used to calculate the sum of ranks per group, required for the calculation of the Kruskal–Wallis *H* statistic. The statistic and corresponding censoring‐adjusted *p* value form the basis for rejection or nonrejection of the null hypothesis. The R code and packages used for the EARS test are provided as Code S1.

The impact of censoring on the ASTs was evaluated by simulating scenarios with varying censoring rates and distributions across groups. This analysis is aimed at determining whether the EARS test′s results remain robust under these conditions. For a detailed description of the simulation study of 1000 cohorts, data generation process, evaluation of clinical cohorts, and statistical methods, please refer to the supporting information. The R code and packages used for the data simulation study are provided as Code S2.

### 2.3. Asymptotic Behavior and Comparison With RMST

To complement the EARS test, RMST analysis was performed using the survRM2 package in R. The truncation time (*τ*) for RMST calculations was determined as the minimum of the largest observed event times across groups, ensuring valid comparisons between arms. The datasets were explicitly designed to violate the PH assumption to evaluate the EARS method′s performance in non‐PH scenarios. This was achieved by introducing a time‐dependent interaction between treatment arms and baseline hazards. Specifically, the hazard rates for one arm were scaled by a factor dependent on both time and baseline hazards, creating nonproportionality. Censoring distributions were varied across groups to explore their influence on test performance. Censoring proportions ranged from 10% to 90%, with censoring times generated using uniform and exponential distributions. The proportion of datasets with *p* < 0.05 was compared, while agreement between methods was defined as the proportion of datasets where both methods either rejected or failed to reject the null hypothesis. Simulations were conducted on 1000 datasets to assess these metrics. Further, to evaluate the asymptotic behavior of the EARS test, additional simulations were performed under the null hypothesis of no group differences. The Kruskal–Wallis test statistic for ASTs was compared to a chi‐squared distribution with *k* − 1 degrees of freedom (*k*: number of groups). The goodness of fit was evaluated by calculating the proportion of simulated test statistics that fell within the expected range of the chi‐squared distribution, providing a measure of agreement.

### 2.4. Theoretical Justification

The EARS test leverages the Kruskal–Wallis test to compare ASTs, which are derived by normalizing observed survival times to the event′s incidence within each group. Specifically, for each group, the survival time of each patient who experiences the event is adjusted by dividing it by the proportion of patients who experience the event within that group. This adjustment ensures that differences in group size or event rates do not unduly influence test outcomes. By modifying the survival times in this way, the EARS test accounts for the unequal event distributions across groups, allowing for a more equitable comparison of survival outcomes. This adjustment is particularly important when group sizes and event rates vary substantially. For example, when one group has a higher event rate than another, it is important that the survival time comparisons reflect not just the timing of events but also the proportion of patients who experience the event. The EARS test corrects for these potential biases, providing a clearer understanding of the differences between groups.

Under the null hypothesis, which assumes no difference between the survival distributions of the groups, the Kruskal–Wallis test statistic follows a chi‐squared distribution with degrees of freedom equal to the number of groups minus one (df = *k* − 1). This ensures the validity of the test for sufficiently large samples. The chi‐squared approximation is robust and reliable when sample sizes are large enough, making the EARS test suitable for applications in clinical research where datasets may be of varying sizes and event rates. The EARS test remains valid even when the data does not adhere to the assumptions of PH or normality, as it is a nonparametric test. This makes it particularly useful in survival analysis, where PH assumptions are often violated or where the data may be skewed or have outliers that would affect parametric tests.

Moreover, by using the Kruskal–Wallis test, the EARS test is able to handle differences in both group size and censoring rates without the need for complex adjustments or assumptions about the underlying distribution of the survival times. This feature makes the EARS test a practical and robust alternative to traditional survival analysis methods, especially when dealing with non‐PH or when specialized software is not available.

## 3. Results

### 3.1. Theoretical Validation and Test Development

To validate the theoretical justification for the EARS test, a simulation study was conducted comparing its results to those of the log‐rank test across 1000 datasets. Each dataset consisted of two groups with varying sizes (50 to 1000 patients per group) and censoring rates randomly assigned between 5% and 75%. Cohort sizes were classified as small (≤ 300 patients), medium (301–700 patients), or large (> 700 patients). Censoring rates were categorized as low (≤ 40%) or high (> 40%), based on the proportion of censored observations in the dataset. Agreement between methods was defined as both tests either rejecting or failing to reject the null hypothesis (*p* < 0.05).

Out of 1000 simulations, 966 cases (96.6%) showed agreement between the EARS and log‐rank tests, while 34 cases (3.4%) differed:

• Small cohorts: one disagreement (5.0%)
• Medium cohorts: nine disagreements (5.2%)
• Large cohorts: 24 disagreements (3.0%)

Censoring rates showed a relatively balanced influence on agreement:

• Low censoring rates: 18 disagreements (3.6%)
• High censoring rates: 16 disagreements (3.2%)

Among the 34 disagreement cases, the median *p* values for the EARS test and log‐rank test were 0.84 and 0.64, respectively. The correlation between EARS *p* values and log‐rank *p* values across all simulations was moderate (*r* = 0.33). The variability in results was illustrated by the distribution of EARS *p* values (mean 0.29, median 0.18, IQR 0.04–0.48) and log‐rank *p* values (mean 0.27, median 0.19, IQR 0.06–0.53; Table 1). Figure S1 displays a scatter plot comparing EARS and log‐rank *p* values, stratified by cohort size, illustrating the correlation between the tests.

**Table 1:** EARS and log‐rank *p* values in simulation study of 1000 cohorts.

	**EARS** **p**	**Log-rank** **p**
Minimum	0.000003	0.000005
1^st^ quartile	0.041643	0.056496
Median	0.183216	0.185655
Mean	0.288153	0.271167
3^rd^ quartile	0.483808	0.430546
Maximum	0.997536	0.995019

Abbreviation: EARS, event‐adjusted rank sum test.

To further assess the reliability of the EARS test, Type I error rates were simulated using datasets with no true differences between groups. The simulation included datasets with two to five groups, where 50% of datasets had two groups and the remaining 50% had three, four, or five groups. The sample sizes ranged from 50 to 1000 patients per group, and censoring rates varied between 5% and 75%. The Type I error rate for the EARS test was 2%, demonstrating that the test does not exhibit an inflated false‐positive rate. In contrast, the Type I error rate for the log‐rank test (log‐rank test for trend when > 2 groups) was lower at 1%, suggesting that the EARS test has a slightly higher tendency to reject the null hypothesis under the null condition.

Statistical power was evaluated by simulating datasets with true differences between groups, maintaining the same distribution of group sizes and censoring rates. The power of the EARS test was 63%, slightly lower than the 68% observed for the log‐rank test. Both tests demonstrated comparable performance in detecting true group differences, indicating that the EARS test is well suited for survival analyses under realistic conditions, including scenarios with more than two groups.

These findings highlight the EARS test′s validity for comparing survival distributions, showing that it performs comparably to the log‐rank test in terms of Type I error control and statistical power.

Next, the characteristics of the EARS test were evaluated using five fictional cohorts comprising 190 to 11160 patients, as detailed in Table 2. Initially, the ASTs for each cohort were plotted and compared between groups (Figure 1). Subsequently, the results of the EARS test were compared to those of the log‐rank, Wilcoxon, Breslow, and Tarone–Ware tests within these five cohorts. All tests rejected the null hypothesis (*p* < 0.05) in Cohorts 2, 3, and 5, whereas no test rejected the null hypothesis in Cohorts 1 and 4 (Figure 2). These results demonstrated the robust performance of the EARS test within these five cohorts, prompting us to proceed with a data simulation study.

**Table 2:** Overview of cohorts used for EARS test development.

**Cohort #**	**1**	**2**	**3**	**4**	**5**
*n*	1000	11 160	1412	190	1000
Median time to event (IQR)	4.1 y (3.9)	1.5 y (2.2)	1.9 y (1.9)	5.3 y (5.5)	3.9 y (4.1)
Median follow‐up, all patients (IQR)	4.8 y (4.2)	2.0 y (2.7)	3.2 y (3.4)	12.5 y (7.7)	4.1 y (4.2)
Median follow‐up, censored (IQR)	2.2 y (3.9)	2.2 y (2.9)	3.8 y (3.5)	13.6 y (4.6)	4.3 y (4.3)

Abbreviations: EARS, event‐adjusted rank sum test; IQR, interquartile range; y, years.

Figure 1Box plots of adjusted survival times (AST) values in 5 cohorts. Each panel corresponds to a single cohort: (a) Cohort 1, Kruskal–Wallis *p* = 0.99; (b) Cohort 2, Kruskal–Wallis *p* = 1.34 × 10^−25^; (c) Cohort 3, Kruskal–Wallis *p* = 2.64 × 10^−34^; (d) Cohort 4, Kruskal–Wallis *p* = 0.40; and (e) Cohort 5, Kruskal–Wallis *p* = 3.07 × 10^−32^. These *p* values are not adjusted for censoring proportions as in the complete EARS test. AST values are presented with whiskers representing the minimum and maximum values in each group. Significant differences are marked with asterisks ( ^∗∗∗∗^ *p* < 0.0001), and “ns” indicates nonsignificant differences. Note that the *y*‐axis scale varies between panels to enhance data visualization.

**Figure:**
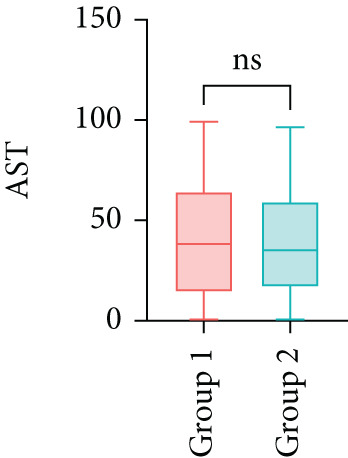
(a)

**Figure:**
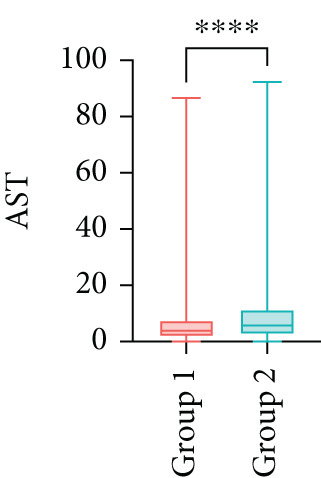
(b)

**Figure:**
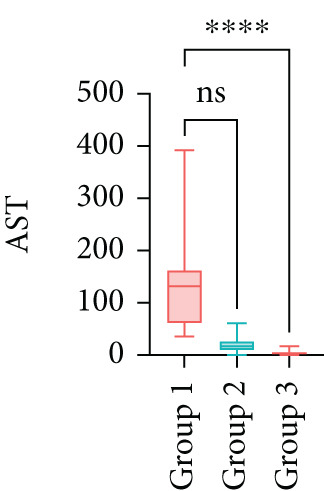
(c)

**Figure:**
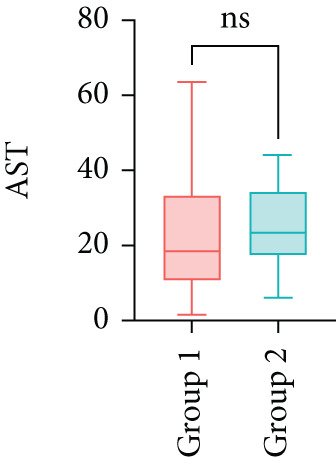
(d)

**Figure:**
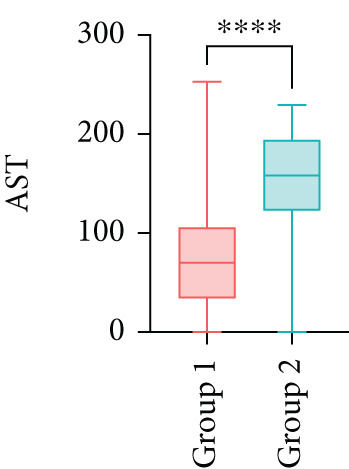
(e)

Figure 2Survival time distribution and comparative analysis with various statistical tests. (a) Bar chart illustrating the number of patients divided by 100 (in pink) and the unadjusted median time to the event of interest (in green) for each of the five cohorts studied during test development. (b) Kaplan–Meier survival curve for Cohort 1, alongside *p* values obtained from the log‐rank, Wilcoxon, Breslow, Tarone–Ware, and event‐adjusted rank sum (EARS) tests. (c) Cohort 2, (d) Cohort 3, (e) Cohort 4, and (f) Cohort 5. Colored areas represent 95% confidence intervals.

**Figure:**
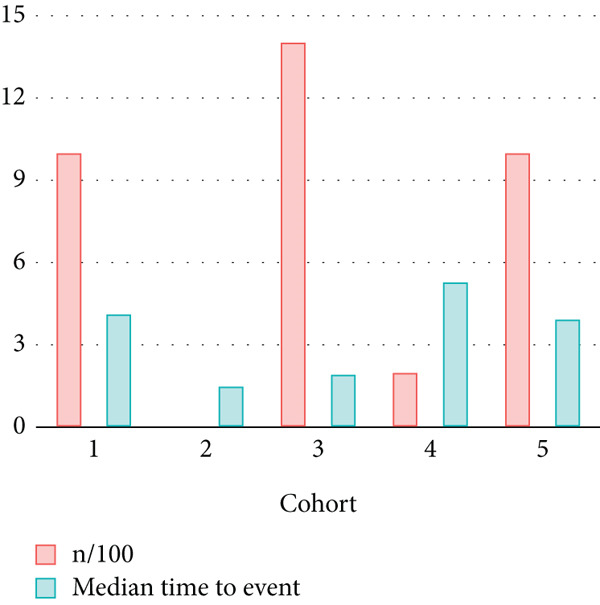
(a)

**Figure:**
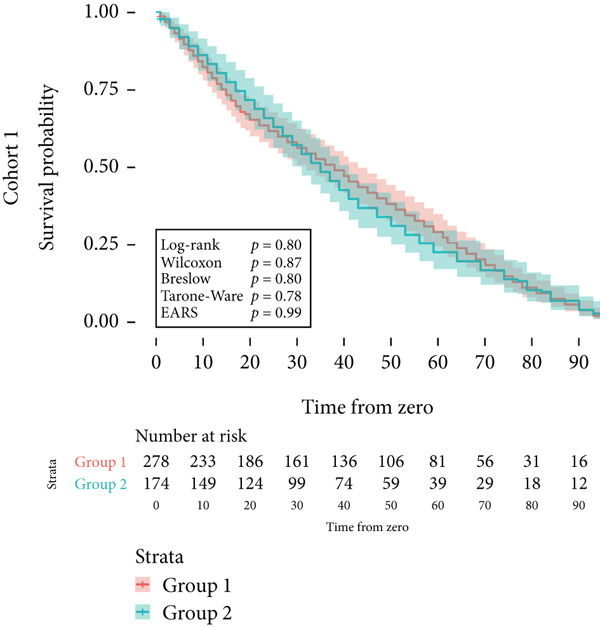
(b)

**Figure:**
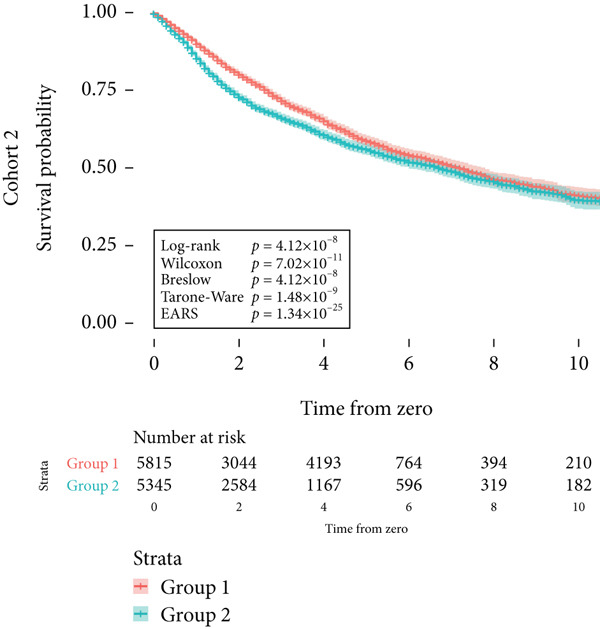
(c)

**Figure:**
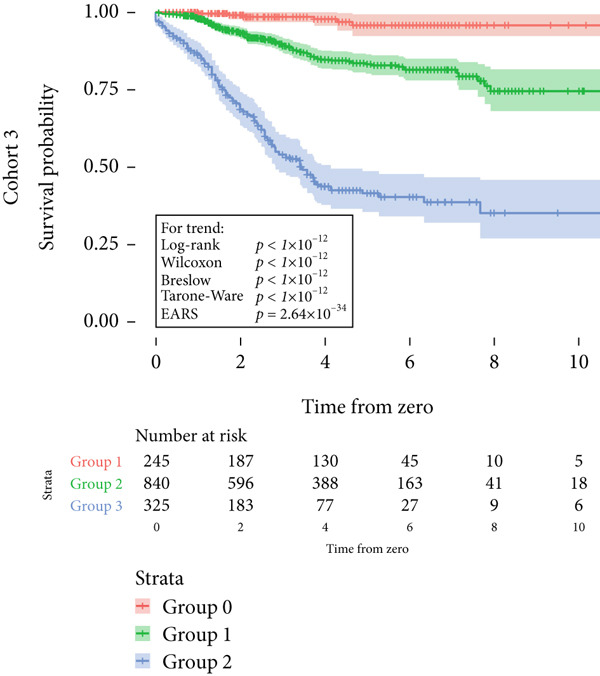
(d)

**Figure:**
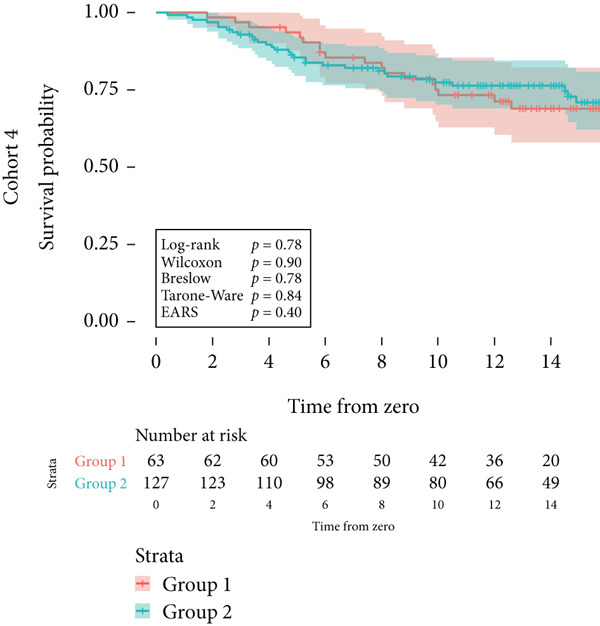
(e)

**Figure:**
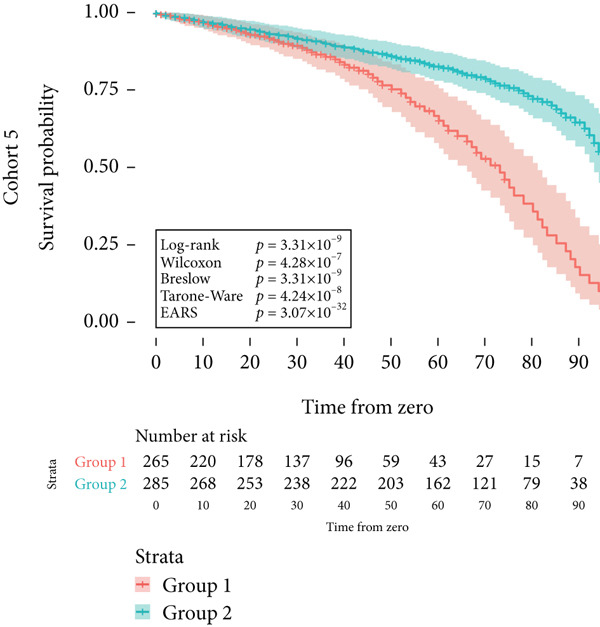
(f)

### 3.2. Asymptotic Behavior and Comparison With Other Non‐PH Tests

In 1000 simulated datasets explicitly designed to violate the PH assumption, the mean *p* value was 0.0308 for EARS, 0.0049 for RMST, 0.0036 for MaxCombo, 0.0043 for the weighted log‐rank test (WLRT, early), and 0.0035 for both WLRT (mid) and WLRT (late). The proportion of cohorts with *p* < 0.05 was 94.4% for EARS, compared to 98.0% for RMST, 98.6% for MaxCombo, 98.3% for WLRT (early), and 98.5% for both WLRT (mid) and WLRT (late). Agreement in rejecting or not rejecting the null hypothesis at *α* = 0.05, when compared to EARS, was 96.2% for RMST and MaxCombo, 96.5% for WLRT (early), and 96.3% for WLRT (late and mid, Table 3). These results indicate that under non‐PH conditions, EARS produces decisions closely aligned with those from other non‐PH methods while yielding slightly higher *p* values on average.

**Table 3:** Summary of EARS and other non‐PH tests in 1000 cohorts.

**Statistic**	**EARS**	**RMST**	**MaxCombo**	**WLRT (early)**	**WLRT (mid)**	**WLRT (late)**
Mean *p*	0.0308	0.0049	0.0036	0.0043	0.0035	0.0035
Median *p*	7.70 × 10^−12^	9.22 × 10^−26^		5.03 × 10^−19^	2.74 × 10^−21^	2.74 × 10^−21^
% *p* < 0.05	94.8	98.4	98.6			
Agreement vs. EARS (%)		96.2	96.2	96.5	96.3	96.3

Abbreviations: EARS, event‐adjusted rank sum test; PH, proportional hazards; RMST, restricted mean survival time; WLRT, weighted log‐rank.

To assess the asymptotic behavior of the EARS test, additional simulations were performed under the null hypothesis of no group differences. The Kruskal–Wallis test statistic for ASTs, as used in the EARS test, approximated a chi‐squared distribution with *k* − 1 degrees of freedom (*k*: number of groups). A *p* value of 0.92 confirmed the goodness of fit, reinforcing the validity of the EARS test for larger sample sizes.

### 3.3. Clinical Cohorts

In the concluding stage of our study, EARS was tested across three distinct clinical cohorts. The first cohort consisted of 1530 patients diagnosed with uveal melanoma, tracked until their demise. Notably, male patients exhibited a higher risk of mortality due to cardiovascular diseases compared to females. This finding was substantiated by the log‐rank test, yielding a *p* value of 0.0038. The EARS test yielded a higher *p* value of 0.0162, as illustrated in Figure 3a.

Figure 3Kaplan–Meier survival curves for clinical cohorts. (a) The first cohort includes 1530 patients, monitored until death. It illustrates a higher mortality risk due to cardiovascular diseases in male patients compared to female patients, as evidenced by all statistical tests of the survival distribution. (b) The second cohort, comprising 80 patients, shows a markedly reduced disease‐specific survival for those with *BAP1*‐mutated uveal melanoma. The EARS test resulted in a higher *p* value than the other tests. (c) The third cohort examines patients born at latitudes below 59°, indicating a poorer disease‐specific survival in uveal melanoma. This is supported by the log‐rank, Wilcoxon, and Breslow tests, whereas the Tarone–Ware and EARS tests did not reject the null hypothesis. The colored areas depict the 95% confidence intervals for each cohort.

**Figure:**
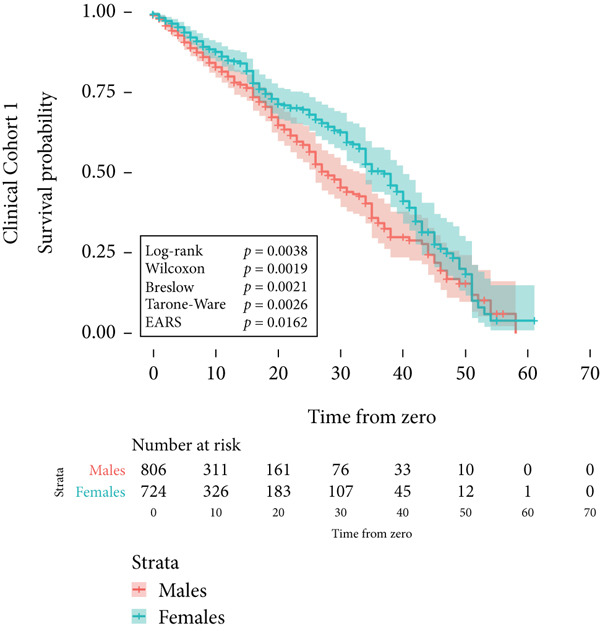
(a)

**Figure:**
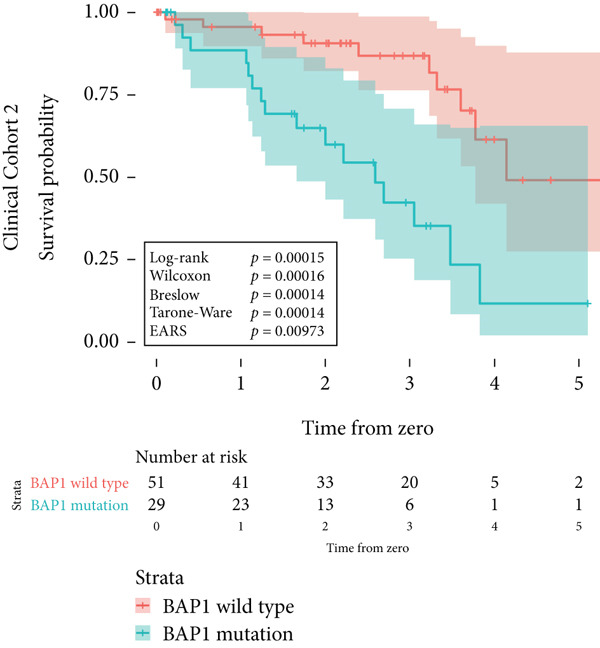
(b)

**Figure:**
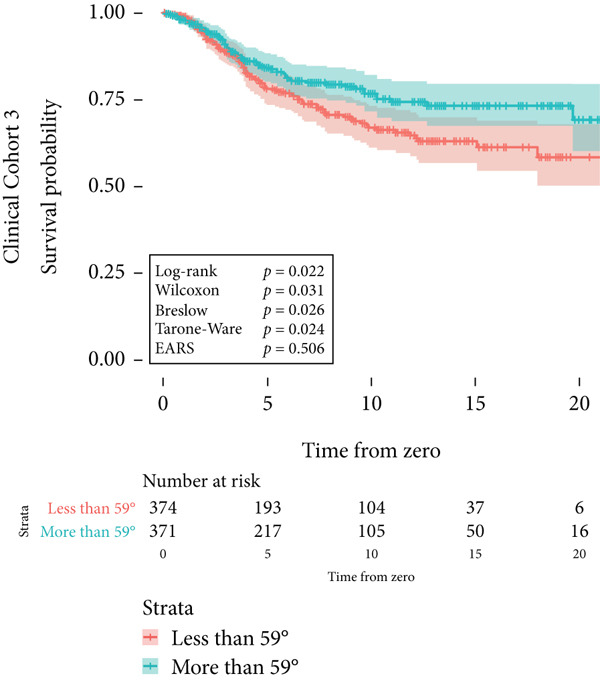
(c)

In the second cohort, which included 80 patients with uveal melanoma, we observed a significantly worse disease‐specific survival in cases of *BAP1*‐mutated uveal melanoma. This was consistently indicated by both the log‐rank and EARS tests, with *p* values of 0.00015 and 0.0097, respectively (Figure 3b). This highlights the prognostic implication of *BAP1* mutations in uveal melanoma.

However, the results from the third clinical cohort presented a contrasting picture. In this cohort, the log‐rank test rejected the null hypothesis that patients born at latitudes lower than 59° did not exhibit worse disease‐specific survival in uveal melanoma compared to those born at or above 59°, evidenced by a *p* value of 0.022. Conversely, the EARS test did not reject the null hypothesis, presenting a *p* value of 0.506 (Figure 3c). The survival curves depicted a graphical divergence, with patients born below 59° tending toward lower survival rates, but with overlapping 95% CIs at all time points. A worked example of the EARS method applied to the largest clinical cohort, a TCGA‐like clinical dataset (Cohort 2), including a Kaplan–Meier plot, key survival metrics, and detailed interpretation, is provided in the supporting information.

## 4. Discussion

Our study introduces the EARS test, offering a novel approach to survival analysis that addresses the challenges posed by non‐PH. The intentionally simplistic calculation of EARS *p* may be appreciated by clinicians, junior researchers, and other nonexperts of survival statistics. The results of our study underscore the utility and practicality of the test in various cohorts.

Our findings indicate that the EARS test provides a simple yet robust approach to survival analysis, including in situations when PH is uncertain or demonstrably violated. In our simulation study of 1000 cohorts, we observed that 966 cases (96.6%) showed agreement in null hypothesis decisions between the EARS and log‐rank tests, while 34 cases (3.4%) differed. This high level of agreement underscores that, although the EARS test and log‐rank test rely on different underlying assumptions, they lead to similar conclusions in most scenarios.

Importantly, our evaluation of Type I error rates demonstrated that the EARS test was close to nominal expectations (2%), whereas the log‐rank test had a somewhat lower rate (1%). Although this indicates that the EARS test may reject the null hypothesis more often under true null conditions, the magnitude of the difference does not appear to be excessively inflated. Moreover, the statistical power of the EARS test (63%) was slightly lower than that of the log‐rank test (68%), indicating broadly comparable performance.

In simulations under non‐PH conditions, we observed that the proportion of cohorts with *p* < 0.05 was 94.4% for EARS compared to 98.0% for RMST, reflecting strong concordance (96.4%) in terms of rejecting or not rejecting the null hypothesis. These simulations also revealed that the censoring rate and distribution had a limited impact on EARS test outcomes, an important consideration for real‐world data analysis. Lastly, the Kruskal–Wallis statistic for ASTs approximated a chi‐squared distribution under the null hypothesis (goodness‐of‐fit *p* = 0.92), aligning with theoretical expectations for large samples.

A key advantage of the EARS test is its accessibility. By performing straightforward calculations of ASTs and then running a standard Kruskal–Wallis test, researchers and clinicians with minimal statistical training can conduct a survival comparison. In situations where specialized survival analysis software is unavailable or when the proportionality of hazards is in question, EARS provides a relatively direct, nonparametric solution. Researchers should, however, still consider established methods—such as time‐varying Cox models, RMST‐based approaches, or other non‐PH tests—depending on study objectives and the nature of the data.

Recently, the “Average Hazard” (AH) framework has been proposed as an alternative effect measure for time‐to‐event outcomes [20, 21]. AH summarizes the event time distribution as a censoring‐free, survival‐weighted incidence rate over a prespecified time horizon and can be expressed in both absolute and relative terms. Extensions to regression models allow covariate adjustment under various censoring assumptions, addressing a limitation of many rank‐based tests. By contrast, the EARS test is a purely nonparametric method focused on hypothesis testing rather than effect size estimation. It does not require specification of a truncation time, parametric model, or covariate set and can be implemented in minimal computing environments. While AH and EARS share the goal of providing interpretable alternatives to the hazard ratio in non‐PH settings, their outputs and intended uses differ: AH provides a magnitude of effect, whereas EARS evaluates whether differences exist in both event incidence and timing. A formal empirical comparison between these approaches, including their statistical power, robustness to censoring, and interpretability, would be valuable future work.

## 5. Limitations

Despite its promise, the EARS test has several limitations. Firstly, there must be at least one event in each group being compared—if one group has no events whatsoever, the test cannot be applied. Secondly, following the conditions for a Kruskal–Wallis test, there should be at least five independent observations for each compared group. Thirdly, while the Kruskal–Wallis test can in principle be sensitive to unequal censoring distributions between groups, the EARS test reduces this influence because the censoring adjustment is applied using the overall censoring proportion in the entire cohort, not per group. Consequently, the *p* value adjustment does not depend on whether censoring rates differ between groups. Only patients who experienced the event are included in the ranking process, so any imbalance in censoring affects results indirectly through the number of events available for ranking, rather than by altering the censoring penalty. Simulation analyses with varying and imbalanced censoring patterns across groups showed only a limited impact on EARS test results. Fourthly, as shown when evaluating clinical cohorts, the EARS test may be overly conservative compared to alternative tests—including log‐rank, Wilcoxon, Breslow, and Tarone–Ware. Fifthly, because our simulation cohorts and fictional datasets may not fully capture clinical complexity, additional evaluations in prospective trials or large‐scale observational studies are needed. Sixthly, the EARS test does not assign ranks to censored patients, so their follow‐up times are not directly included in the Kruskal–Wallis statistic. However, censored patients do influence the results in two ways: (i) They are included when calculating the event proportion in each group, which forms the denominator for ASTs, and (ii) they contribute to the overall censoring proportion used in the *p* value adjustment. This design simplifies the method and facilitates its use in spreadsheet software, but it also means that precensoring survival time from censored cases is not explicitly ranked. While this represents a potential loss of information, simulations with censoring proportions as high as 90% still showed close agreement between EARS and other established non‐PH tests. Seventhly, the current implementation of the EARS test does not provide a mechanism for adjusting survival comparisons for additional covariates such as age, sex, or comorbidities, which is often desirable in clinical studies. Potential approaches to incorporate covariates could include applying a rank‐based analysis of covariance (ANCOVA) to the ASTs, using matched cohorts, or using stratified EARS analyses where comparisons are made within subgroups defined by the covariates of interest. Alternatively, residuals from a regression model that adjusts for covariates could be substituted for the raw ASTs in the ranking procedure. These extensions may enhance the utility of EARS in multivariable contexts but require further methodological development and validation.

## 6. Conclusion

In conclusion, the EARS test emerges as a valuable addition to available methods for analyzing survival data when PH is violated or cannot be assumed. Its ability to capture both timing and proportion of events in a straightforward manner, coupled with broad software accessibility, should make it attractive to researchers lacking advanced statistical training. At the same time, users need to be cognizant of its assumptions and practical constraints, including the overall (rather than per‐group) penalty for censoring. By sharing the code and Excel templates, we encourage wider adoption and further validation of the EARS test in varied clinical and research contexts.

## Ethics Statement

All experiments were performed in accordance with relevant guidelines and regulations. Data from real‐world patients were collected from anonymized public datasets. According to the Swedish Ethical Review Act, the requirement for ethical approval is waived if no personal or sensitive information is collected or processed, if no interventions are planned, if no biological materials are processed, and if the method does not aim to affect individuals, neither physically nor psychologically, and does not entail any risk of harm for the research subjects [18].

## Conflicts of Interest

The author declares no conflicts of interest.

## Funding

This study was funded by the Karolinska Institutet, 10.13039/501100004047, FS‐2021‐0010, and Region Stockholm, 20200356.

## Supporting information


**Supporting Information** Additional supporting information can be found online in the Supporting Information section. In addition to the Supporting Information Excel file (AST.xlsx) and the raw data for all five test development cohorts (Cohorts 1 to 5.xlsx), as described in the “Data Availability Statement” section, we provide a Supporting Information.pdf. This PDF contains Codes S1 and S2, a description of the simulation study and data generation process, an overview of the clinical cohorts, the statistical tests and analyses, and Figure S1.

## Data Availability

In addition to Code S1, which offers the R code necessary for implementing the EARS test and details the utilized R packages, and the raw data for all five cohorts used for test development, we have developed a Supporting Information Excel file (AST.xlsx) tailored to simplify the computation of EARS *p* values. Accompanied by a sample dataset, this file is structured to allow users to substitute it with their own data, covering the variables “Group,” “Time,” and “Event.” Once user data is inputted, the Excel file is programmed to automatically calculate the AST for each patient. The data can be copied into one of the many openly available online software for the Kruskal–Wallis test or into statistical software. The EARS test is also available as an R package, downloadable from GitHub at https://github.com/gustavstalhammar-ui/EARS.
