# Supplementary material for: Introducing the Event‐Adjusted Rank Sum (EARS) Test: A Simple Approach to Survival Analysis Independent of Proportional Hazards
Source: Biomed Res Int. 2025 Nov 18;2025:2142254. doi: 10.1155/bmri/2142254 (PMC12624226; doi:10.1155/bmri/2142254)

Supplementary material

Introducing the Event-Adjusted Rank Sum (EARS) Test: A Simple Approach to Survival Analysis Independent of Proportional Hazards

Gustav Stålhammar, M.D., Ph.D.[1,2]

[1]Ocular Oncology Service and St. Erik Ophthalmic Pathology Laboratory, St. Erik Eye Hospital, Stockholm, Sweden.
[2]Department of Clinical Neuroscience, Division of Eye and Vision, Karolinska Institutet, Stockholm, Sweden.

Contents

Supplementary code 1. 2

Simulation study 4

Data generation process 4

Clinical cohorts 4

Statistical tests and analyses 5

Supplementary code 2. 6

Supplementary Figure 1. 9

Worked example 10

Supplementary code 1.

R code used for the EARS test.

```
# Read the data file, which should have the following three columns: Group, Time, and
Event.
# Replace "Cohort 1.csv" with the name of your file. Adjust the separator (sep) if needed.
bmt <- read.csv("Cohort 1.csv", sep = ";")

# Function to calculate modified times for EARS analysis
calculate_modified_time <- function(df) {
  for (g in unique(df$Group)) {
    group_proportion <- sum(df$Group == g & df$Event == 1) / sum(df$Group == g)  # Event
proportion
    df$ModifiedTime[df$Group == g & df$Event == 1] <- df$Time[df$Group == g & df$Event
== 1] / group_proportion
  }
  df$ModifiedTime[df$Event != 1] <- NA  # Assign NA to censored data
  return(df)
}

# Function to adjust P-value by censoring rates
adjust_p_value_by_censoring <- function(pval, df) {
  censoring_proportion <- mean(df$Event != 1)  # Proportion of censored patients
  adjustment_factor <- 1 - censoring_proportion  # Non-censoring proportion
  adjusted_pval <- pval / adjustment_factor  # Adjusted P-value
  return(adjusted_pval)
}

# Calculate modified times
bmt <- calculate_modified_time(bmt)

# Filter out rows where ModifiedTime is NA (i.e., censored data)
result <- subset(bmt, !is.na(ModifiedTime))

# Perform Kruskal-Wallis test
kruskal_test <- kruskal.test(ModifiedTime ~ Group, data = result)

# Adjust the P-value based on the censoring proportion
adjusted_p_value <- adjust_p_value_by_censoring(kruskal_test$p.value, bmt)

# Calculate median times (modified and actual)
modified_medians <- aggregate(ModifiedTime ~ Group, data = result, median, na.rm =
TRUE)
actual_medians <- aggregate(Time ~ Group, data = bmt, median, na.rm = TRUE)

# Combine results into a single table
final_result <- merge(modified_medians, actual_medians, by = "Group")
names(final_result) <- c("Group", "Modified Median", "Actual Median")

# Print results
print(final_result)
print(paste("Kruskal-Wallis Statistic:", kruskal_test$statistic))
```

```
print(paste("Original P-Value:", kruskal_test$p.value))
print(paste("Adjusted P-Value:", adjusted_p_value))
```

Simulation study

A simulation study was designed to assess the agreement between the EARS test and the Log-rank test in survival analysis through a simulation of 1000 cohorts. The key parameters for the cohort generation and the rationale behind their selection are detailed below:

1. Number of patient samples: The simulation consisted of 1000 distinct cohorts. This sample size was chosen to ensure a robust statistical analysis, allowing for a comprehensive evaluation of the tests' agreement across a variety of scenarios.
2. Cohort size (*n*): Each cohort's size was randomly set between a minimum of 25 and a maximum of 1000 patients. This range was selected to reflect realistic variations in cohort sizes typically encountered in clinical studies and to test the robustness of the EARS and Log-rank tests across different sample sizes.
3. Number of groups: The cohorts were structured to contain between 2 and 5 patient groups, randomly determined. This variation in group numbers was intended to simulate real-world clinical studies.
4. Follow-up time: The time to event or last follow-up for each patient was randomly set between 0 and 100. This broad range was chosen to mimic the variable follow-up periods seen in longitudinal studies and to assess the tests' performance over both short and extended observation times.
5. Event coding: The event of interest (*e.g.*, death, disease progression, complications after surgery) was coded as '1', while all other outcomes (including patients alive at last follow-up, losses to follow-up and any other event than the event of interest) were coded as '0'.

Data generation process

For each cohort, data were generated using a randomization approach, following these steps:

1. Cohort size determination: The number of patients in each cohort was randomly selected within the defined range to ensure variability.
2. Group assignment: Patients within each cohort were randomly assigned to a group, with the total number of groups per cohort varying between 2 and 4.
3. Survival time simulation: Each patient's survival time was generated following a uniform distribution between 0 and 100 to simulate real-world survival data variably.
4. Event status simulation: The event status for each patient (event occurred or censored) was randomly determined, aligning with typical survival analysis datasets where the event and censored cases are randomly distributed.

Clinical cohorts

In the final phase, the EARS test was validated using three additional real-world clinical cohorts, collectively comprising 2355 patients. We acquired anonymized time-to-event data from these cohorts (on February 12–14, 2024), available in the public domain as reported in their respective original publications.[18-20] The author had no access to information that could identify individual participants during or after data collection.

The first clinical cohort included 1530 patients diagnosed consecutively with uveal melanoma, as documented in a comprehensive 2023 study assessing causes of mortality.[19] For the purpose of EARS validation, we specifically compared the risk of dying from cardiovascular diseases between female and male patients, examining gender as a potential risk factor.

In the second cohort, 80 patients with uveal melanoma were selected from The Cancer Genome Atlas.[18] This cohort was part of a broader study that evaluated a wide array of genetic markers. During the EARS validation, we focused on comparing

patients with BAP1-mutated melanomas to those harboring the wild-type variant of the gene, as identified through DNA sequencing. The mutation status of BAP1 is widely recognized as a significant prognostic factor in uveal melanoma.

Lastly, the third cohort comprised 745 patients with uveal melanoma, originating from a study that explored the potential prognostic impact of latitude on the disease.[20] This evaluation compared Swedish patients born at latitudes above 59 degrees with those born at or below this latitude. Although latitude serves as a prognostic indicator, its relative prognostic significance is less than that associated with tumor size, *BAP1* mutation status, monosomy 3 and other strong prognostic markers.

Statistical tests and analyses

For each cohort, the *P* values from both the EARS and Log-rank tests were computed. A significance threshold (α) of 0.05 was used. Cohorts were classified as 'rejections' if the *P* value from either test was below this threshold.

To assess the agreement between the EARS and Log-rank tests, the following analyses were conducted:

1. Tracking rejections: The number of cohorts in which the EARS and Log-rank tests agreed (either both rejected or both did not reject the null hypothesis) was counted.
2. Compilation of results: A comprehensive data frame (results_df) was created, compiling the *P* values from both tests for all cohorts.
3. Statistical metrics:
   - Concordance (Pearson correlation) between the *P* values from EARS and Log-rank tests was calculated to assess linear correlation.
   - Cohen's Kappa statistic was computed to quantify the level of agreement between the tests, correcting for agreement that could occur by chance. The Kappa statistic was interpreted as:
     - Kappa < 0: No agreement
     - Kappa between 0.00 and 0.20: Slight agreement
     - Kappa between 0.21 and 0.40: Fair agreement
     - Kappa between 0.41 and 0.60: Moderate agreement
     - Kappa between 0.61 and 0.80: Substantial agreement
     - Kappa between 0.81 and 1.00: Almost perfect agreement.[21]

The use of these statistical measures were aimed to provide a dual perspective: The Pearson correlation offered insights into the linear relationship between the *P* values, while Cohen's Kappa provided a measure of agreement taking into account the possibility of chance correlation.

Supplementary code 2.

R code used for the the simulation study.

```
# Load necessary libraries
library(survival)
library(dplyr)

# Set a seed for reproducibility
set.seed(123)

# Define the number of cohorts and other parameters
num_cohorts <- 1000
min_patients <- 25
max_patients <- 1000
min_groups <- 2
max_groups <- 5

# Create an empty list to store the results
results_list <- list()

# Function to generate random data for a cohort
generate_cohort_data <- function() {
  cohort_size <- sample(min_patients:max_patients, 1)
  num_groups <- sample(min_groups:max_groups, 1)

  Group <- sample(0:(num_groups - 1), cohort_size, replace = TRUE)
  Time <- runif(cohort_size, 0, 100)
  Event <- sample(0:1, cohort_size, replace = TRUE)  # Generate 0s and 1s for events

  df <- data.frame(Group, Time, Event)
  return(df)
}

# Function to calculate the EARS statistic
calculate_ears_statistic <- function(df) {
  # Function to calculate modified time
  calculate_modified_time <- function(df) {
    for (g in unique(df$Group)) {
      group_proportion <- sum(df$Group == g & df$Event == 1) / sum(df$Group == g)
      df$ModifiedTime[df$Group == g & df$Event == 1] <- df$Time[df$Group == g & df$Event
== 1] / group_proportion
    }
    df$ModifiedTime[df$Event != 1] <- NA  # Assign NA to censored data
    return(df)
  }

  df <- calculate_modified_time(df)
  result <- subset(df, !is.na(ModifiedTime))

  # Perform Kruskal-Wallis test
  kruskal_test <- kruskal.test(ModifiedTime ~ Group, data = result)
```

```
  # Adjust P-value by censoring rates
  censoring_proportion <- mean(df$Event != 1)
  adjustment_factor <- 1 - censoring_proportion
  adjusted_p_value <- kruskal_test$p.value / adjustment_factor

  return(adjusted_p_value)
}

# Function to calculate the Log-rank statistic
calculate_logrank_statistic <- function(df) {
  logrank_test <- survdiff(Surv(Time, Event) ~ Group, data = df)
  logrank_p_value <- pchisq(logrank_test$chisq, df = 1, lower.tail = FALSE)
  return(logrank_p_value)
}

# Create counters to track rejection counts
ears_rejections <- 0
logrank_rejections <- 0
concurrent_rejections <- 0

# Loop to generate cohorts, calculate EARS and Log-rank statistics, and track results
for (i in 1:num_cohorts) {
  cohort_data <- generate_cohort_data()
  ears_p_value <- calculate_ears_statistic(cohort_data)
  logrank_p_value <- calculate_logrank_statistic(cohort_data)

  if (ears_p_value < 0.05) {
    ears_rejections <- ears_rejections + 1
  }

  if (logrank_p_value < 0.05) {
    logrank_rejections <- logrank_rejections + 1
  }

  if (ears_p_value < 0.05 && logrank_p_value < 0.05) {
    concurrent_rejections <- concurrent_rejections + 1
  }

  results_list[[i]] <- data.frame(EARS_P_Value = ears_p_value, Logrank_P_Value =
logrank_p_value)
}

# Combine all cohort results into a single data frame
results_df <- do.call(rbind, results_list)

# Calculate summary statistics for EARS_P_Value and Logrank_P_Value
summary_stats <- summary(results_df[c("EARS_P_Value", "Logrank_P_Value")])

# Extract the desired statistics
mean_values <- c(mean(results_df$EARS_P_Value), mean(results_df$Logrank_P_Value))
median_values <- c(median(results_df$EARS_P_Value),
median(results_df$Logrank_P_Value))
min_values <- c(min(results_df$EARS_P_Value), min(results_df$Logrank_P_Value))
```

```
max_values <- c(max(results_df$EARS_P_Value), max(results_df$Logrank_P_Value))
q1_values <- c(quantile(results_df$EARS_P_Value, 0.25),
quantile(results_df$Logrank_P_Value, 0.25))
q3_values <- c(quantile(results_df$EARS_P_Value, 0.75),
quantile(results_df$Logrank_P_Value, 0.75))

# Create a table with summary statistics
summary_table <- data.frame(
  Statistic = c("Mean", "Median", "Min", "Max", "1st Quartile", "3rd Quartile"),
  EARS_P_Value = c(mean_values[1], median_values[1], min_values[1], max_values[1],
q1_values[1], q3_values[1]),
  Logrank_P_Value = c(mean_values[2], median_values[2], min_values[2], max_values[2],
q1_values[2], q3_values[2])
)

# Print the summary table
print(summary_table)

# Create a function to generate the contingency table
generate_contingency_table <- function(ears_p_values, logrank_p_values, alpha_threshold
= 0.05) {
  ears_rejected <- ears_p_values < alpha_threshold
  logrank_rejected <- logrank_p_values < alpha_threshold

  contingency_table <- table(EARS_Rejected = ears_rejected, Logrank_Rejected =
logrank_rejected)

  return(contingency_table)
}

# Call the function with your results
contingency_table <- generate_contingency_table(results_df$EARS_P_Value,
results_df$Logrank_P_Value)

# Print the contingency table
print(contingency_table)

# Calculate Pearson correlation and p-value
correlation_result <- cor.test(results_df$EARS_P_Value, results_df$Logrank_P_Value,
method = "pearson")

# Extract correlation and p-value
correlation <- correlation_result$estimate
p_value <- correlation_result$p.value

# Create a table with Pearson correlation and p-value
correlation_table <- data.frame(
  Pearson_Correlation = correlation,
  P_Value = p_value
)

# Print the correlation table
print(correlation_table)
```

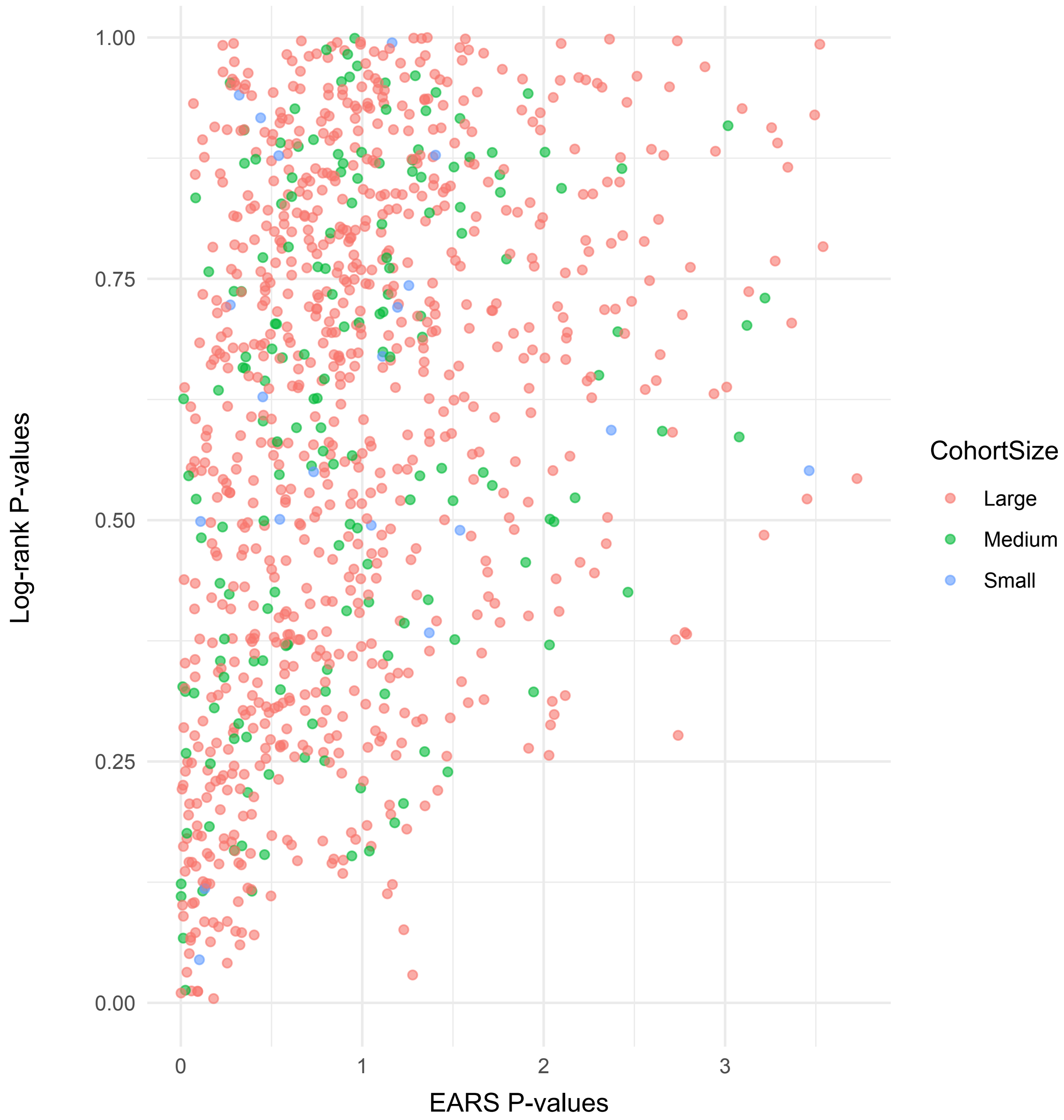

Supplementary Figure 1.
Scatter plot comparing EARS and Log-rank *P*-values, stratified by cohort size, illustrating the correlation between the two tests. Each dataset consisted of two groups with sizes randomly selected between 50 and 1000 patients per group, and censoring rates ranged between 5% and 75%. Cohort sizes were categorized as small (≤ 300 patients, blue), medium (301–700 patients, green), or large (> 700 patients, red). The x-axis represents EARS *P*-values, while the y-axis shows Log-rank *P*-values, providing a visual representation of the relationship and agreement between the two methods across 1000 simulated datasets.

Worked example

As a worked example, we applied the EARS test to Cohort #2, which included 11,160 patients from a TCGA-like clinical dataset divided into two groups:

Group 0 (*n*=5,345) experienced 1,841 events (34.4% event proportion; 65.6% censored) with a median survival of 6.25 years (95% CI, 5.65–6.94) and survival probabilities of 85.7%, 65.4%, and 54.8% at 1, 3, and 5 years, respectively.

Group 1 (*n*=5,815) had 1,784 events (30.7% event proportion; 69.3% censored), a median survival of 6.90 years (95% CI, 6.43–7.44), and survival probabilities of 90.2%, 71.3%, and 57.9% at 1, 3, and 5 years. The EARS test yielded a Kruskal–Wallis χ^2 statistic of 115.76 with a $P=1.65\times10^{-26}$, consistent with the difference in survival distributions suggested by the Kaplan–Meier curves (log-rank $P<0.0001$).

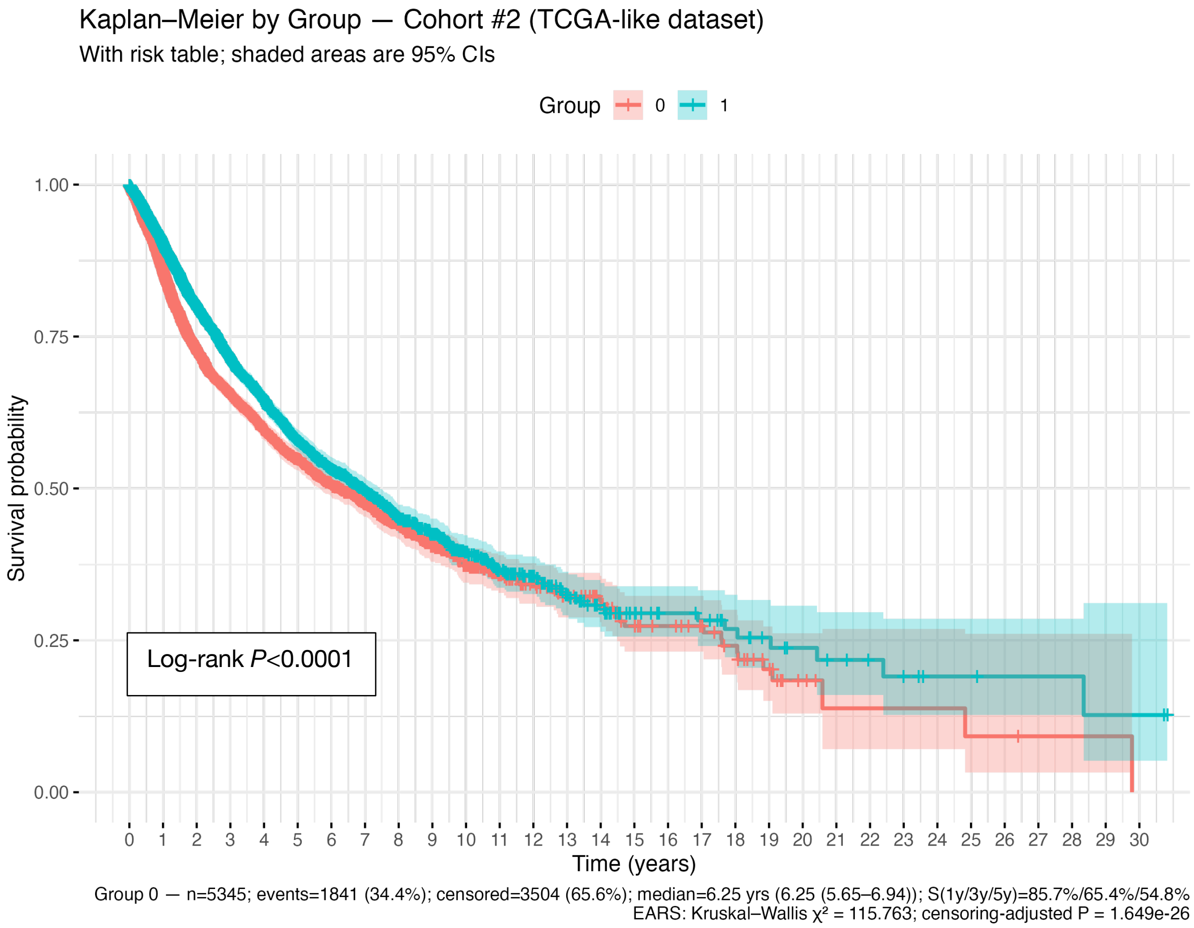

Supplement: Supplementary file 1 — Supporting Information Additional supporting information can be found online in the Supporting Information section. In addition to the Supporting Information Excel file (AST.xlsx) and the raw data for all five test development cohorts (Cohorts 1 to 5.xlsx), as described in the “Data Availability Statement” section, we provide a Supporting Information.pdf. This PDF contains Codes S1 and S2, a description of the simulation study and data generation process, an overview of the clinical cohorts, the statistical tests and analyses, and Figure S1. [file BMRI-2025-2142254-s001.zip › Supplementary Material.pdf]
